# MGcount: a total RNA-seq quantification tool to address multi-mapping and multi-overlapping alignments ambiguity in non-coding transcripts

**DOI:** 10.1186/s12859-021-04544-3

**Published:** 2022-01-14

**Authors:** Andrea Hita, Gilles Brocart, Ana Fernandez, Marc Rehmsmeier, Anna Alemany, Sol Schvartzman

**Affiliations:** 1 Epigenetics unit, Diagenode s.a., Liège, Belgium; 2 Department of Biology, Humboldt-Universität zu Berlin, Berlin, Germany; 3 Department of Anatomy and Embryology, Leiden University Medical Centre, Leiden, The Netherlands

**Keywords:** RNA-seq, Non-coding, Small RNA, Alignment, NGS, Quantification, Counting, Multi-mapping, Multi-overlapping, Single-cell, Map equation

## Abstract

**Background:**

Total-RNA sequencing (total-RNA-seq) allows the simultaneous study of both the coding and the non-coding transcriptome. Yet, computational pipelines have traditionally focused on particular biotypes, making assumptions that are not fullfilled by total-RNA-seq datasets. Transcripts from distinct RNA biotypes vary in length, biogenesis, and function, can overlap in a genomic region, and may be present in the genome with a high copy number. Consequently, reads from total-RNA-seq libraries may cause ambiguous genomic alignments, demanding for flexible quantification approaches.

**Results:**

Here we present Multi-Graph count (MGcount), a total-RNA-seq quantification tool combining two strategies for handling ambiguous alignments. First, MGcount assigns reads hierarchically to small-RNA and long-RNA features to account for length disparity when transcripts overlap in the same genomic position. Next, MGcount aggregates RNA products with similar sequences where reads systematically multi-map using a graph-based approach. MGcount outputs a transcriptomic count matrix compatible with RNA-sequencing downstream analysis pipelines, with both bulk and single-cell resolution, and the graphs that model repeated transcript structures for different biotypes. The software can be used as a python module or as a single-file executable program.

**Conclusions:**

MGcount is a flexible total-RNA-seq quantification tool that successfully integrates reads that align to multiple genomic locations or that overlap with multiple gene features. Its approach is suitable for the simultaneous estimation of protein-coding, long non-coding and small non-coding transcript concentration, in both precursor and processed forms. Both source code and compiled software are available at https://github.com/hitaandrea/MGcount.

**Supplementary Information:**

The online version contains supplementary material available at 10.1186/s12859-021-04544-3.

## Background

Next Generation Sequencing (NGS) experiments have become the gold standard for many applications within the transcriptomics field, including gene expression profiling, novel transcript discovery and allele diversity detection [1, 2]. Advanced library preparation methods enable researchers to sequence and analyze RNA from individual cells [3–5] and to infer cell differentiation trajectories, recognize rare cell populations and identify transcription regulatory mechanisms [6].

While early NGS experiments focused on the detection of polyadenylated RNA (i.e., messenger RNA [mRNA] and polyadenylated long non-coding RNA [lncRNA]), later RNA library preparation methods made it possible to target small regulatory RNAs (small RNAs) [7–9] and also full transcriptomes (hereafter referred to as total-RNA-seq). Total-RNA-seq simultaneously captures polyadenylated RNA and non-polyadenylated RNA, which together include all types of mRNA, lncRNA, and small RNA, both as precursors and in processed forms. With total-RNA-seq library preparation methods recently having reached single-cell resolution [10–14], it has become possible to investigate transcriptional regulation through non-coding RNA with unprecedented detail.

### Challenges in total-RNA quantification

**Fig. 1:**
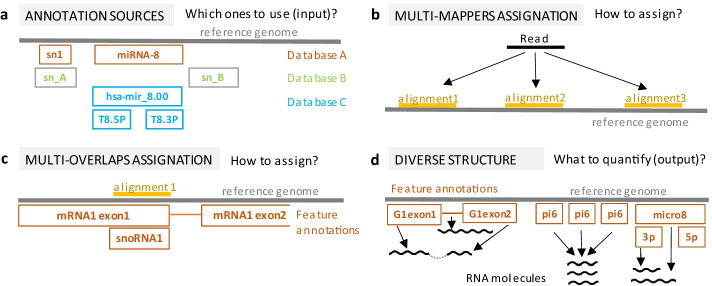
Main challenges in total-RNA-seq quantification. **a** Integration of databases from multiple sources (indicated in orange, green and cyan) can lead to redundant annotations of the same feature with different names, formats or slightly different coordinates. **b** Example of a multi-mapping read (black line) that aligns equally well to more than 1 position (yellow lines) and cannot be assigned to a genomic origin unequivocally. **c** Example of a multi-overlapping read that maps to a genomic position where two annotated features coexist (a mRNA exon and a snoRNA) and cannot be assigned to a feature unequivocally. **d** Examples of the heterogeneous relations between genomic loci and transcribed molecules integrated in total-RNA-seq: a cluster of small-RNA loci (e.g. pi6, from the piRNA biotype) can actively transcribe the same product simultaneously; microRNA transcripts result in two distinct transcripts (e.g. unprocessed mi8 is post-clipped to 3p/5p mature forms); long molecules comprising exons and introns undergo splicing, resulting in reads from precursor and mature transcripts

Figure 1 introduces the four main challenges in quantifying the output of total-RNA-seq experiments. First, different databases annotate transcribed genomic regions under different structures. For example, Ensembl, Gencode and Refseq [15–17] store formatted annotations under protein-coding structure (gene, transcript, exon), while biotype specialized databases as miRbase [18] annotates precursor and mature forms of microRNAs. Furthermore, less extensively studied RNAs such as piRNAs are annotated in specialized databases only. As a consequence, total-RNA-seq analysis needs to integrate multiple databases, to homogenize formats and to deal with redundant annotations with possibly miss-matching coordinates (Fig. 1a).

Second, reads frequently align to more than one annotated feature (ambiguous alignments). These reads comprise multi-mappers, which align to multiple genomic locations (Fig. 1b), and multi-overlappers, which align to a genomic location with multiple annotated features (Fig. 1c). When quantifying protein-coding feature expression, a frequent approach is to discard multi-mapping and multi-overlapping reads, since they usually occur at low proportion. A downside of this approach is that transcripts that overlap in the same genomic region, or that as a result of gene recombination, transposition or duplication events have a high copy number, will be underrepresented in the final counts. This fact becomes particularly relevant when simultaneously quantifying transcripts from different non-coding RNA classes.

Different strategies have been proposed to quantify multi-mappers and multi-overlappers, reviewed in [19]. Raw counting tools such as featureCounts can count all alignments, fractionally count all alignments or randomly select one alignment [20, 21]. Rescue methods such as CoCo prioritize features with more uniquely-mapping alignments, assuming that these agglomerate in active loci and that multi-mappers result from partial sequence overlap with inactive loci [22, 23]. Probabilistic approaches such as RSEM, Kallisto and Salmon statistically weight transcript or isoform candidates, and are more suitable for quantifying well-characterized transcriptomes [24–26]. In small-RNA quantification, algorithms consider neighboring patterns around each multi-mapping alignment [27, 28]. Mmquant reports multi-mappers as merged gene counts [29], and GeneQC employs Machine Learning to provide the user with uncertainty estimates for ambiguous alignments [30].

Finally, biotype-specific pipelines quantify expression levels by taking into account the genomic structure of the biotype in question. This raises a conceptual question: what is the feature output level at which transcript abundance estimation is most meaningful for total-RNA-seq? (Fig. 1d). While protein-coding feature abundance is usually summarized at isoform or gene level, small-RNA tools quantify at transcript level [31–35] and, in some cases, they can collapse expression from transcripts arising from multiple genomic regions into one . In this regard, total-RNA-seq analysis demands for a flexible approach that adaptively defines feature quantification output levels suiting all RNA biotypes, independently of the available annotations.

Taken together, simultaneous quantification of small RNA, lncRNA and mRNA requires new strategies that simultaneously account for the diverse nature of each transcript, without relying on assumptions that could lead to biotype-dependent quantification biases.

### MGcount

Motivated by the above, we developed a novel RNA-seq quantification approach named MGcount (Multi-Graph count). MGcount handles multi-overlapping reads that arise from small RNAs originating from within long-RNA exons or introns, takes into account both polyadenylated and non-polyadenylated reads from long RNA, assigns multi-mapping reads with heterogeneous profiles, and defines output expression levels in an adaptive data-driven manner. MGcount is:

- Generalizable to the simultaneous quantification of multiple RNA biotypes
- Compatible with any genome and annotations set
- Compatible with single-cell data
- Available as a single command-line program

We generated and analysed RNA-seq libraries from 4 well-studied species, namely, *A. thaliana, H. sapiens, M. musculus* and *C. elegans* to characterize the behaviour of multi-mapping and multi-overlapping reads and the effect of MGcount and other quantification tools (CoCo, RSEM, featureCounts and mmquant) over distinct classes of RNA. We further evaluated MGcount’s performance in two different human RNA templates (K562 cell line and human brain) and validated the accuracy of the estimated counts for a set of 20 small-RNA markers with RT-qPCR. Finally, we tested MGcount on a publicly available total-RNA-seq dataset with single-cell resolution. [14].

## Implementation

### Algorithm

**Fig. 2:**
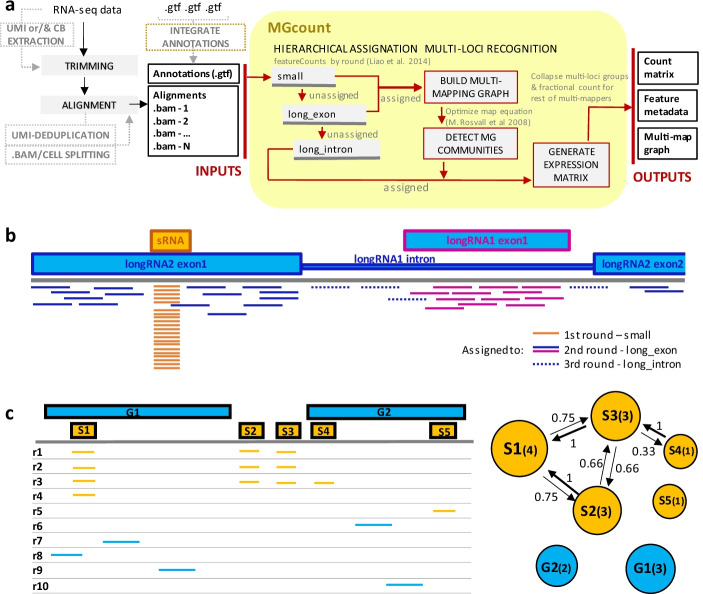
MGcount strategy. **a** MGcount takes a set of genomic alignments (BAM files) and a GTF RNA feature annotations file as inputs. The algorithm assigns reads hierarchically and then models multi-mapping assignments in a graph using the Rosvall’s map equation [36, 37]. As output, MGcount provides an RNA expression count matrix (where feature communities are collapsed as new defined features), a feature metadata table and the graphs. **b** Illustration of how the hierarchical assignation can resolve multi-overlappers: reads that map to small-RNA and long-RNA features are assigned to small-RNA in the first round; reads that map to long-RNA introns and long-RNA exons are assigned to long-RNA exons in the second round; remaining reads are assigned in the last round. **c** Illustration of multi-mapping small-RNA and long-RNA exon graphs generation by MGcount. Reads ri (i = 1, 10) have been hierarchically assigned to $S_{1}, S_{2}, S_{3}, S_{4}, S_{5}$ (small-RNA biotypes, yellow), and $G_{1}, G_{2}$ (long-RNA biotypes, blue). Each vertex in the directional multi-mapping graphs (right) corresponds to a feature and has a size proportional to the logarithm of the number of alignments. Edges connect vertices with common multi-mapping reads, with weights proportional to the number of common multi-mappers normalized by the total number of alignments of the source vertex. Hence, the weight of the edge connecting S1 with S2 becomes 3/4 (reads mapping both S1 and S2 divided by reads aligned to S1). (CB: Cell Barcode, UMI: Unique Molecular Identifier)

MGcount starts with a set of genomic alignments of RNA-seq reads (one BAM file per sample/cell) and a set of RNA feature annotations stored in a single gene transfer format (GTF) file. To quantify RNA features abundance, MGcount employs two strategies, summarized in Fig. 2. First, a hierarchy based on transcript body length is used to solve multi-overlapping ambiguities across RNA biotypes during alignment-to-feature assignment. Second, communities of sequence-related RNA features are detected, and defined as new aggregated features. MGcount is built on top of featureCounts [21], a computationally efficient counting software.

#### Hierarchical assignation

MGcount hierarchically assigns reads to annotated genomic features in three pre-defined sequential rounds, named “small”, “long_exon” and “long_intron”. First, in the “small” round, alignments are assigned to small-RNA biotypes (such as microRNA, piRNA, snRNA, snoRNA, tRNA, YRNA and vaultRNA) and thereby prioritized in situations where an alignment overlaps with a small-RNA embedded within a long-RNA (mRNA or lncRNA) feature (Fig. 2b). As we show in Additional file 1, a–d, these constitute the majority of overlapping cases. This is justified by the length disparity between small and long RNAs. A read overlapping both a small-RNA and a long-RNA feature will have more likely been generated from a small RNA than a much longer long-RNA transcript when reads cluster on the small RNA. In cases where reads are present throughout the long-RNA locus and when most or all reads might have come from the long RNA, assigning overlapping reads to the small RNA will impact the expression quantification of the long RNA only marginally. The second and third rounds in the hierarchy assign alignments to long-RNA exons and long-RNA introns, respectively. These hierarchies are justified since unspliced transcripts (with introns) are short-lived compared to mature transcripts (without introns). Hence, it is more likely that a mature transcript is detected in situations where an exon of a long RNA overlaps with an intron of another long RNA.

The list of biotypes included in each round can be customly expanded or modified by the user. In addition, each round can be configured through five arguments (Table 1), which facilitates simultaneously dealing with small-RNA and long-RNA features annotated with different formats and under different columns in the GTF annotation file. The five arguments are (1) “feature”, the annotation type considered for alignment-to-feature assignation; (2) “feature_output”, the annotations attribute for which feature abundance will be reported; (3) “feature_biotype”, the annotations attribute defining the biotype assigned in the corresponding round; (4) “min_overlap”, the minimum overlapping fraction of the read required to assign an alignment to an annotation; and (5)“ml_flag”, to activate or disable communities detection and feature aggregation for each round (see next section).

**Table 1:** Configurable parameters and default values for each read-to-feature assignation round

Round	Feature	Feature_output	Feature_biotype	Min_overlap	ml_flag
Small	Transcript	Transcript_name	Transcript_biotype	1	True
Long_exon	Exon	Gene_name	Gene_biotype	1	True
Long_intron	Gene	Gene_name	Gene_biotype	1	True

Note that long-RNA introns are by default defined by the full gene body coordinates

#### Multi-mapper communities detection

MGcount exploits graph structures to model resemblances between annotated features, in its potential to produce the same transcript, from real evidence coming from RNA-seq data. MGcount builds a directed weighted graph G = (V, E) where each vertex from the set of vertices V is a feature (as defined by the feature_output parameter), with a weight equal to the log-transformed number of assigned alignments (Fig. 2c). Directional edges (E) connect features that share multi-mapping reads, with a weight proportional to the ratio of multi-mapping reads between the two connected vertices, normalized by the total number of reads assigned to the source vertex. Graphs are generated independently for small and long RNA, using the full pool of alignments from all input samples.

Next, highly related features, where reads systematically multi-map, are grouped together in communities by minimizing an objective function known as the map equation [36, 37]. The map equation formulates the theoretical limit to compress the description of an infinite random walk trajectory along the graph, tagging vertices to describe within-community movement and tagging communities to describe inter-community movement with codes of bits. The goal is to minimize the description length (total number of bits) as a function of the communities. This occurs when grouping densely connected vertices in communities where the random walk stays the maximum within and moves the minimum between. Resultant communities, hereafter referred to as MG communities, represent groups of features with the potential to produce identical or nearly identical RNA transcripts. Each MG community is given an identifier that is subsequently used to aggregate the corresponding alignments.

In the small-RNA graph, although MGcount creates one graph for the total set of features, MG communities are independently detected per biotype. For long RNA, the top represented biotype is assigned to an MG community when this contains features from different biotypes. However, when a community contains both pseudogene and non-pseudogene features, a preference is made for the latter, in order to prioritize for the active biotypes.

#### Count matrix building

MGcount generates one expression matrix for each hierarchical assignation round and concatenates them in a single output matrix. For each read, each alignment first gets a “fractionated count” of 1/N, where N is the number of multi-mappers or multi-overlappers that survived the hierarchical assignment because they aligned to two features from biotypes in the same round. Next, counts for annotations that have been aggregated together in a community by the map equation are summed up (communities become newly defined features). In this way, the systematic ambiguity in multi-mapping reads collapses into a single MG community while the remaining signal is reported as fractionated counts.

### Software execution

#### Inputs and outputs

MGcount requires three inputs: a TXT file listing the paths to the BAM input files, a GTF file, and the output directory path. Additional optional arguments specify whether the data is from single or paired sequencing, whether the library preparation has been done in stranded or unstranded manner and assignation rounds configuration (Table 1). Further configuration arguments and usage description is provided in the software user-guide available in the MGcount Github repository (https://github.com/hitaandrea/MGcount).

At the end of its execution, MGcount provides the following outputs:

- A count matrix where each row corresponds to a feature as defined by feature_output (either single features or MG communities aggregating several features) and each column corresponds to one input BAM file
- A feature metadata table reporting: feature names matching row names in the count matrix, the counting round of hierarchical assignation, and its configuration parameters, a flag designing whether a feature belongs to an MG community, and the feature biotype
- A sparse adjacency matrix for each multi-mapping graph generated (small RNA and/or long RNA), stored as a symmetric, integer, squared matrix. Each matrix element stores the number of alignments that multi-map to a pair of features (defined by row and column), and the diagonal contains the total number of alignments per feature.
- A table of MG communities linking each original feature in the GTF file with the resultant count matrix and metadata feature identifiers. It includes both unique features (which remain unmodified) and aggregated features (which are collapsed following MG communities). Also, the table stores the total number of alignments per feature.

#### Accessibility

The source code is available in the MGcount Github repository (https://github.com/hitaandrea/MGcount). MGcount is fully written in Python3. The software can be installed using the “pip” command and run as a python3 module. Alternatively, the MGcount software can be downloaded as a single, binary, compiled file and executed independently of Python. This binary has been compiled to run in the Ubuntu operating systems. To run MGcount, featureCounts [21] is required.

#### Integrated annotations

The scope of the MGcount quantification is bounded by the features annotated in the reference GTF file. To maximize the scope of the analysis, we combined annotations from DASHR, RNAcentral, miRbase and Ensembl [15, 18, 38, 39] in a single GTF file. The MGcount repository provides integrated GTF annotations for human, arabidopsis, mouse and nematode, and the corresponding R scripts used for their generation. These can be used as a template to integrate annotations from other species.

## Results

### Hierarchical assignation resolves small-RNA long-RNA multi-overlappers

In order to assess the potential impact of overlapping features from different biotypes on RNA-seq analysis, we explored their overlap frequencies. For this task, we used as a reference the customized GTF file that integrate several databases (see previous section) for the following species: *H. sapiens*, *M. musculus*, *C. elegans* and *A. thaliana* (Additional file 1, a-d). We observed in all organisms that most overlaps take place between long-RNA and small-RNA transcripts, which significantly differ in transcript body length. These results support the rationale for the two-step hierarchical function in MGcount.

**Fig. 3:**
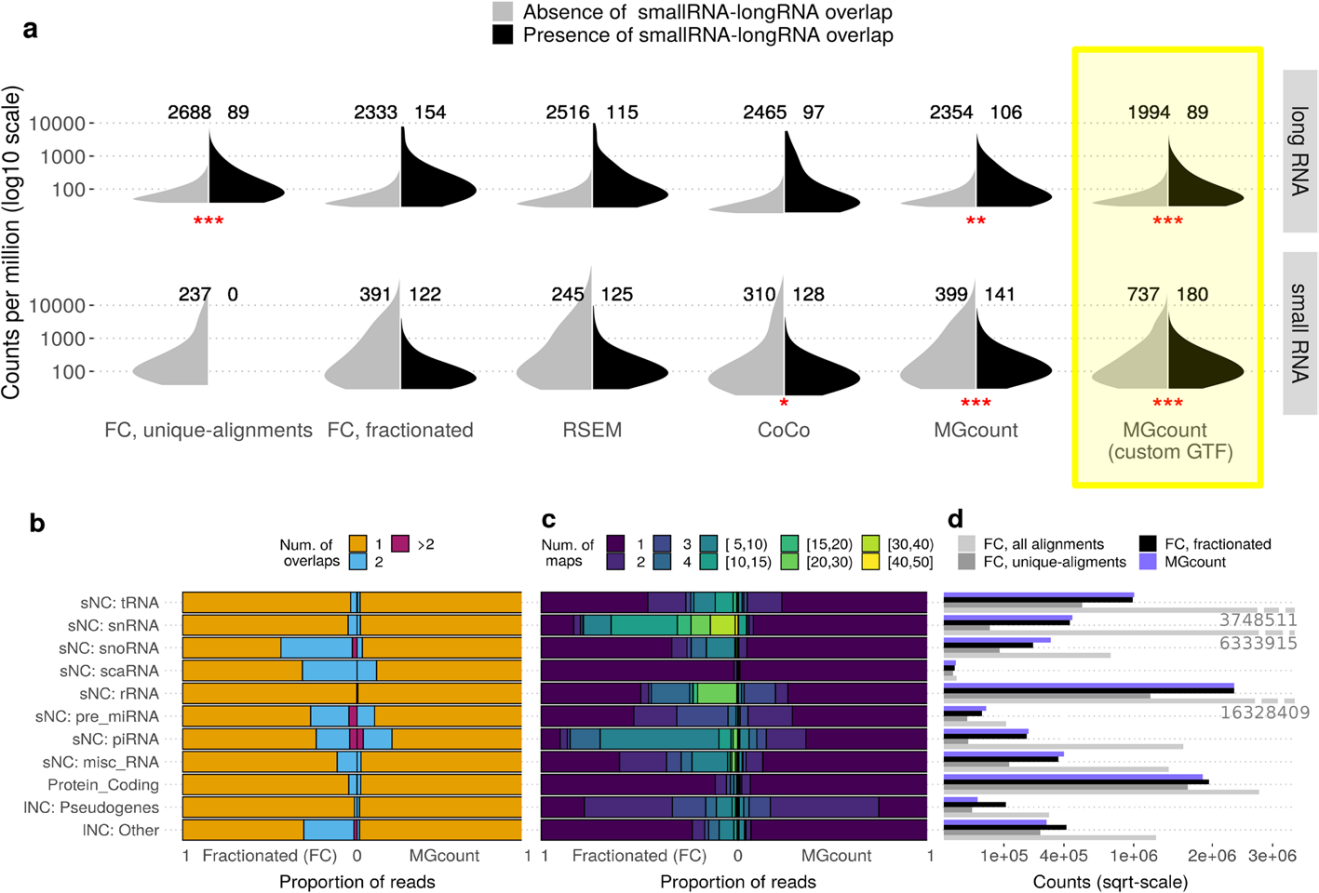
Hierarchical assignation evaluation and multi-mapping and multi-overlapping patterns quantification. **a** Distributions of counts per feature obtained with different quantification strategies in presence and absence of small RNA-long RNA overlaps for long RNA and small RNA features (FC: featureCounts). The 3000 features with highest counts are included in the distributions (including small and long RNA), and their amount is indicated above each distribution. Red stars correspond to the *p* value of a TOST test (0–0.001: ***, 0.001–0.01: **, 0.01–0.05: *) with equivalence margins at 0.35 log10-normalized counts per million (CPM). **b** Proportion of reads from a human total-RNA-seq library overlapping to 1, 2 or more annotations according to fractionated alignments assignation (left) and after MGcount assignation by hierarchical rounds (right). **c** Proportion of reads multi-mapping to a given number of genomic locations (up to 50), according to fractionated alignments assignation (left) and after MG community aggregation by MGcount (right).** d** Comparison of counts when only uniquely-mapping reads are counted; all alignments are counted, all alignments are fractionally counted as 1 divided by the number of multi-assigments or quantified with MGcount (HBR: Human Brain, sNC: small non-coding; lNC: long non-coding))

Next, we evaluated the effect of the hierarchical assignation of reads on resolving small-RNA long-RNA multi-overlapping ambiguities. For this, we hypothesized that the level of expression of a given transcript is not influenced by the fact that other transcripts can originate from the same locus. For example, mRNAs can be expected to have a similar count distribution, whether they overlap with a small RNA or not. We compared the count distributions from the two pooled human brain libraries, for long-RNA and small-RNA features, and in presence or absence of small-RNA long-RNA overlaps (Fig. 3a). Assuming the expression of a transcript is independent of its overlapping condition with other features, the two distributions of counts should be similar. We tested for the equivalence of the distributions using the Two-One-Sided t-Tests (TOST), on different algorithms in gene-level mode, for the top 3000 expressed genes: featureCounts with only inclusion of unique-alignments and with fractionated count of ambiguous alignments, RSEM, CoCo, and MGcount (communities mode disabled) with the Ensembl GTF. Additionally we tested MGcount with the customly integrated GTF.

For long RNA, significantly similar distributions were obtained when hierarchically assigning reads with MGcount and considering only uniquely-mapping reads with featureCounts. This suggests that multi-overlapping alignments are mainly associated with small-RNA reads and that these two strategies are both adequate to quantify long-RNA biotypes. Fractionated count of multi-overlappers with featureCounts led to an inflated estimation of transcript abundance, due to the incorrect fractionated assignment of reads that originated from small-RNA transcripts to their embedding long RNA. Results from CoCo and RSEM show a slight increase of counts for the overlapping transcripts which again can be explained by the miss-assignment of a few small RNAs.

For the quantification of small RNA, considering only reads aligned to a unique annotation resulted in discarding small-RNA transcripts embedded within (i.e., fully overlapping with) a long RNA. MGcount and CoCo produced significantly similar distributions. Remarkably, the use of MGcount with a custom GTF integrating annotations from other sources than Ensembl showed that missing annotations can have a strong impact in transcript quantification due to miss-assignment of multi-overlappers originated from unannotated small RNAs.

In summary, the hierarchical assignment strategy from MGcount was the one producing the most equivalent count distributions with and without overlapping events for both long-RNA and small-RNA features. This supports the rationale that reads that fully map to a small RNA that overlaps a long RNA most likely belong to the small RNA, and demonstrates that MGcount’s hierarchical assignation approach can successfully resolve long-RNA small-RNA overlapping ambiguities.

### MGcount reduces multi-mapping and multi-overlapping ambiguity

To investigate the scope of ambiguous alignments by biotype, we computed the fraction of multi-mappers and multi-overlappers for each biotype in total-RNA-seq libraries from human brain (Fig. 3b, c left), mouse liver, arabidopsis roots and nematodes (Additional file 1, e–f), using featureCounts. The majority of multi-overlapping reads resulted from small-RNA loci embedded within larger long-RNA loci, as observed in Additional file 1 a-d. Alignments exhibiting triple overlaps resulted mostly from reads mapping to regions where a short small RNA arises from a middle-sized small RNA simultaneously embedded within a long RNA. This agrees with the biogenesis pathways of snoRNA-derived microRNAs and piRNAs [40–43]. Multi-mapping reads originated mostly from small RNA loci, with high numbers of genomic positions. This can be explained by the small RNAs’ high copy numbers in the genome, which are largely due to retrotransposition events [44]. We observed a particularly prominent effect in mid-sized small RNAs such as tRNA and snRNA, which links to its nature and sequence repetition [45, 46]. The same holds for a small subset of snoRNA where we observed a portion of reads mapping to 10–15 genomic regions while most snoRNA reads map uniquely. This owes to snoRNA diversity: while some snoRNAs are produced from a unique locus, others have been found to be encoded in tandem copies [47, 48]. With consistency between species (Additional file 1, e–f), the observed patterns highlight the magnitude of the biological signal encoded in reads mapping to more than one RNA feature. Figure 3b, c (right) show how multi-feature alignment ambiguity fractions are reduced with MGcount: due to the hierarchical assignment strategy, multi-overlappers occur less frequently, and the multi-graph strategy collapses features where reads systematically multi-map, converting them into single features. Additionally, by integrating annotations into communities, more single-annotated features are detected since the multi-mapping signal is collapsed as opposed to rescuing methods (CoCo) or probabilistic methods (RSEM) (Additional file 1, k). Communities also facilitate back-trace of multi-mapping reads in case of interest. Our results show that a direct relationship exists between multi-mapping or multi-overlapping patterns and the biological nature of different transcripts, demonstrating the need for an adaptive strategy that treats multi-mapping as an aggregated signal rather than as an ambiguity or an artifact.

Figure 3d and Additional file 1, g show the impact of different raw counting rules (discard ambiguous alignments, count all ambiguous alignments, fractionally count ambiguous alignments, or MGcount) in the total counts per biotype. The quantification of protein-coding genes is little impacted by the counting method given lower multi-mapping situations. However, for non-coding features while discarding multi-mappers and multi-overlappers results in a loss of information, including them all inflates substantially their expression values. Fractionated counting and MGcount force that the total contribution of a read is 1. However, MGcount reduces ambiguity compared to raw fractionated counting (Fig. 3c, d) through the hierarchical assignation and the aggregation of MG communities. The reduction of pseudogene counts is explained because several pseudogenes are assigned to other biotypes while aggregating MG communities within the MGcount algorithm. Besides, larger counts are obtained for small-RNA biotypes as a consequence of the hierarchical assignation step (Fig. 2b). Altogether highlights the sensitivity of RNA expression quantification to the ambiguous alignments handling approaches when dealing with non-coding data.

### Multi-mapping graphs capture RNA locus structure and identify feature communities coding for sequence-similar transcripts

**Fig. 4:**
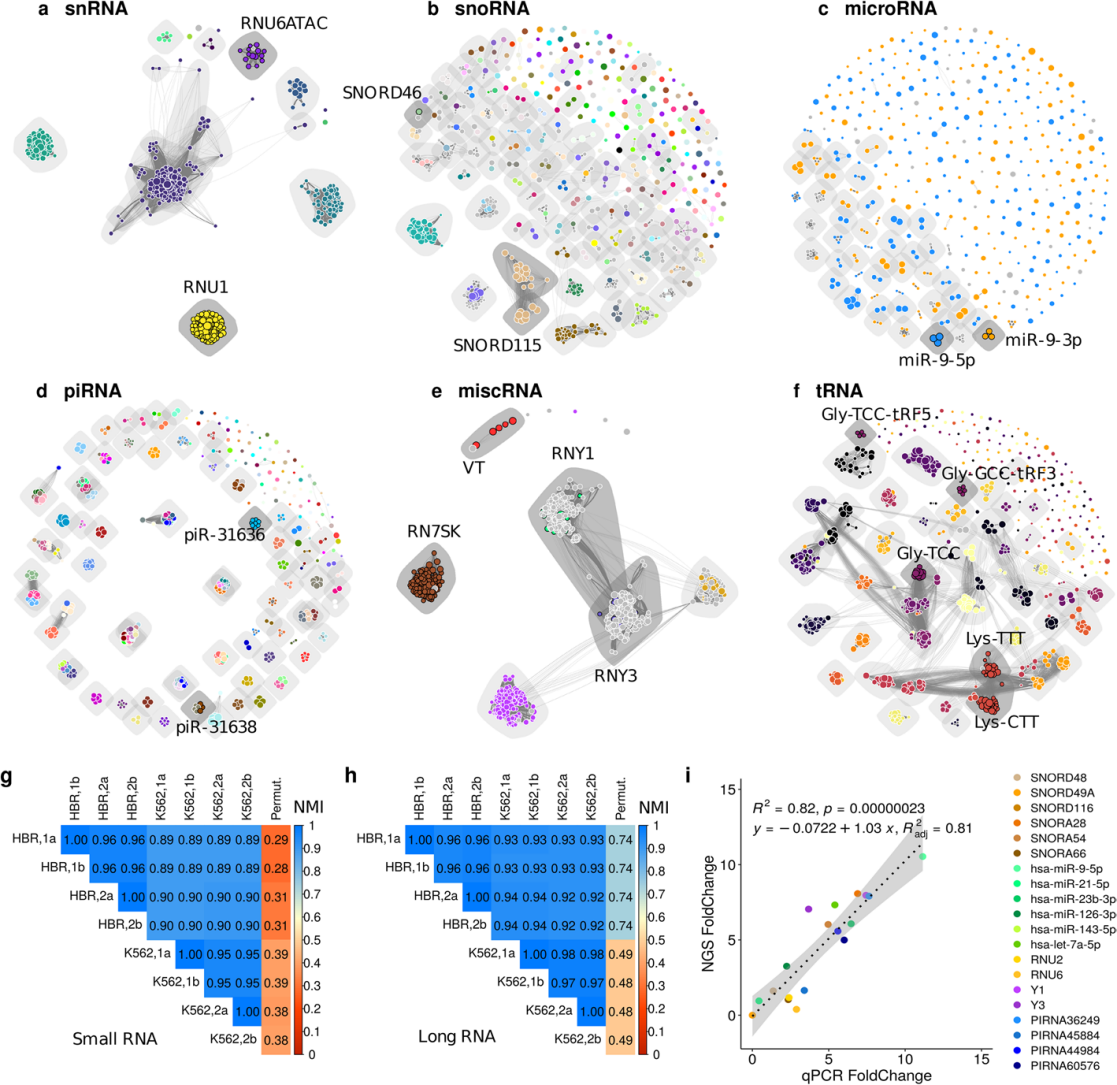
Graphs of small-non coding main biotypes in human libraries. Each vertex is an annotated features with size proportional to its number of aligned reads and color representing its gene symbol in relation to the Hugo Gene Nomenclature Committee (HGNC) (annotations not following HGNC are colored in gray and often correspond to computationally predicted annotations). Each edge connects two features with shared multi-mappers with thickness proportional to the fraction of shared multi-mappers over the total alignments. Shared grey areas delineate MG communities. **a** snRNA graph. Vertices are colored by first number after “RNU” pattern in transcript symbol. **b** snoRNA graph. Vertices are colored by first number after “SNORNA” pattern in transcript symbol. **c** microRNA graph. Vertices are colored by “3p” (orange) and “5p” (blue) patterns in transcript symbol. **d** piRNA graph. Vertices are colored by first number after “PIRNA” pattern.** e** miscRNA graph. Vertices are colored separately by YNY1, YNY3, YNY4, RN7SK, RN7SL, VT patterns in transcript symbol.** f** tRNA graph. Vertices are colored by amino-acid 3-letters reference pattern in transcript symbol. **g–h** Matrices comparing the similarity between MG communities obtained from different input data or algorithm runs using the the Normalized Mutual Information (NMI) index. Last column in each panel shows comparison with a random permutation of the cluster labels. **i** Linear regression fit (black dashed line) and its 95% confidence level interval (gray area) modelling the relationship between RT-qPCR and NGS fold change estimation of Human Brain and K562 expression for 20 small-RNA markers of different biotypes. Linear regression fit (black dashed line) and its 95% confidence level interval (gray area) modelling the relationship between RT-qPCR and NGS fold change estimation of human brain and K562 expression for 20 small-RNA markers of different biotypes. (HBR: Human Brain)

Figure 4 explores the sub-graphs generated by MGcount for the most abundant small-RNA biotypes obtained from the pool of human libraries (K562 cell line and human brain). In general, our results show very different sub-graph topologies for different biotypes. In most of the cases, we detected that features grouped together in each MG community have similar annotations as given by the Hugo Gene Nomenclature Committee (HGNC), which establishes a standard nomenclature framework for RNA classes within major small-RNA biotypes in human [49, 50]. For example, the snRNA graph has a few large MG communities that correspond to the different spliceosome components, present in multiple copies [51]. This is also the case for miscRNAs, which comprise various RNA biotypes such as 7SKRNA or YRNA sub-units. MGcount automatically detected MG communities for 7SKRNAs and YRNAs and their respective retrotransposition-derived pseudogenes spread over the entirety of the genome [52]. Most microRNA annotations did not form MG communites, with the exception of a few loci that code for the same microRNA. These consistently aggregated by corresponding mature forms (3p or 5p). We observed more heterogeneous profiles with snoRNAs, for which large and small communities were detected. The family of piRNAs exhibited compact communities in relation with genomic regions where piRNA sequences reside [53]. For tRNA-derived fragments, communities mainly followed amino acid type and fragment position within the tRNA precursor.

These results demonstrate that MGcount can successfully detect small-RNA communities in a biotype-specific manner and suggests that it will be useful in species with poor annotations. Furthermore, some MG communities integrated features that did not follow the HGNC annotations system and which were associated with computationally predicted annotations (grey nodes in Fig. 4) with other well- characterized small RNAs. For example, SNORD46 (Chr1:44,776,492-44,776,589) was clustered together with AC009365.1 (Chr7:132,753,023-132,753,126), a repeated locus diverging in only 15 out of 104 nucleotides. This suggests a potential application of MGcount in assigning computational predictions to their corresponding RNA families during quantification.

Additional file 1, h–i displays three subgraphs from the long-RNA graph extracted by randomly sub-sampling 500 MGcount features out of those newly defined by MGcount. These were either features that had remained single, or newly aggregated features as communities of multiple, originally annotated features. MGcount defined 2951 long-RNA MG communities in our libraries, while 26,060 features remained single. Large communities detected by MGcount often aggregated pseudogenes with protein-coding features, showing that MGcount successfully avoids attributing read counts to pseudogene inactive loci copies by aggregating pseudogenes with the active gene in a community.

### MG community detection is robust at both intra- and inter-sample levels

To evaluate the robustness of the MG community detection, we compared the communities detected with different seeds (for random-number generators) on the same and on different total-RNA-seq input datasets. We separately processed each replicate of human brain and K562 libraries twice and computed the Normalized Mutual Information (NMI) between partitions of commonly clustered features (Fig. 4g–h). Each solution was also compared to randomized MG communities obtained by permuting the grouping labels of all vertices in the graphs. Long-RNA and small-RNA partitions were nearly identical for the two different runs within the same input dataset (NMI = 1), demonstrating stable convergence and minimal variability due to the algorithm’s stochastic component. Communities across biological replicates showed high similarity in both human brain and K562 libraries, and a comparison between the two templates exhibited only a small reduction of the similarity of solutions. In summary, our analysis showed high reproducibility between MG communities obtained from RNA-seq libraries prepared under similar technical conditions, independent of the RNA origin, and demonstrates the robustness of MGcount’s community detection.

### Validation of expression quantification via RT-qPCR

To independently check the accuracy of the quantification between small RNAs of different biotypes, we compared the total-RNA-seq MGcount expression levels of human brain and K562 libraries with estimates of expression levels from RT-qPCR for 20 small-RNA markers with different multi-loci profiles. All transcript abundances were normalized by SNORD49A, which was highly expressed in both samples. The linear regression showed good adjustment in modelling the fold-change concentration relationship between human brain and K562 libraries, independently measured with total-RNA-seq libraries and with RT-qPCR (Fig. 4i).

### MGcount detects expression of cell-specific non-coding RNA communities

**Fig. 5:**
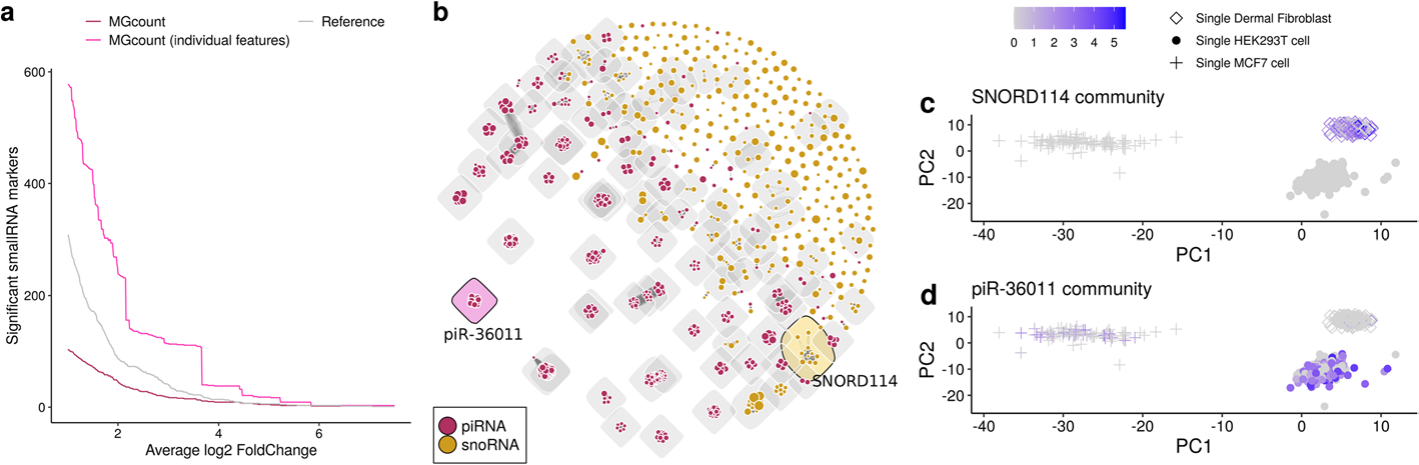
MGcount quantification outputs in a single-cell case example dataset. **a** Number of statistically significant features (*p* value below 0.05) detected by log2 fold-change average. **b** MGcount sub-graph for snoRNAs and piRNAs where SNORD114 and piR-36011 communities are highlighted. **c** Expression level of SNORD114 community by cell represented in the PC1-PC2 space. **d** Expression level of piR-36011 community by cell represented in the PC1-PC2 space. (PC: Principal Component)

In order to evaluate its performance at single-cell resolution, we ran MGcount on a public single-cell total-RNA-seq dataset [14] consisting of 637 cells from three human cell-lines (dermal fibroblasts, HEK293T cells and MCF-7 cells). We performed differential expression analysis on small-RNA features between the three cell populations (Wilcoxon Rank Sum test, adjusted *p* value below 0.05) using count tables in which features were aggregated into MG communities by MGcount and count tables obtained with the reference pipeline described in [14]. In both cases, we used the GTF file that integrates several databases. The average log2 fold-change of differentially expressed small-RNA features detected by MGcount was larger than that of the reference pipeline, indicating that the aggregation of the multi-mapping signal helps detecting stronger effects. With the reference pipeline, we detected 397 statistically significant small-RNA-annotated features at an average log2 fold-change above 0.5, whereas with MGcount, we detected 179 features (out of which 94 were communities of multiple, originally annotated features, and 85 were individual features) (Fig. 5a). These correspond to 1167 of the originally annotated features. Remarkably, by setting the log2 fold-change threshold at 2.5, MGcount detects 28 significant features (including 10 MG communities). These equals to 132 features while with the reference pipeline only 63 are detected. Some MG communities were predictive markers of specific cell types, e.g., the SNORD114 loci tandem cluster located within the human 14q32 locus for dermal fibroblasts (log2 fold-change: 4.52) and the piR-36011 cluster for HEK293T cells (log2 fold-change: 2.33) (Fig. 5b–d). These results demonstrate that MGcount recovers biologically meaningful information from multi-assigned reads at single-cell resolution.

## Discussion

RNA-seq reads frequently align to multiple places in the genome or to genomic regions that encode more than one transcript. Traditional RNA-seq pipelines commonly discard such reads, which does generally not pose a problem in the quantification of protein-coding transcripts. However, the amount of biological information encoded in ambiguous alignments in datasets with non-coding RNA can be considerable (Fig. 3b–d). While a number of solutions for the assignation of ambiguously mapping reads to the expression of corresponding RNA features have been proposed, these solutions focus on particular biotypes or model species; premises are not fully met by all total-RNA-seq datasets, which demand flexible approaches to the simultaneous quantification of any transcript.

Here, we propose a flexible quantification framework to interrogate heterogeneous RNA-seq datasets comprising different non-coding RNA biotypes. First, a hierarchical assignation workflow resolves overlaps of small RNAs embedded in long-RNA loci and allows to distinctly quantify spliced and unspliced features of long RNA. Then, to quantify reads that map to multiple locations in the genome (“multi-mappers”), families of features with almost identical sequences are automatically detected and aggregated in MG communities. With this approach, we gain confidence that the given read originated from a community of annotations rather than a single genomic locus. This approach defines a meaningful output level for the quantification of different biotypes in a data-driven manner and collapses repeated loci that are associated with the same RNA product. This solution may also be used to quantify poorly annotated transcripts as a community (e.g., RNY1 in Fig. 4e) instead of diluting them as several “unknown features”, each of which at a low level of expression. We believe MGcount preserves the multi-mapping information for downstream analyses, improving the quantification of small-RNA biotypes and long RNAs with duplicated sequences, and reducing assignation errors and biases associated with multi-alignment handling premises that do not suit all biotypes. The concept of gene merging has previously been suggested for the study of mRNA [29]. However, these approaches result in the same gene being included in different merged-gene groups, as we observed for mmquant (Additional file 1, k). Here, we propose a graph-based approach that allows to distinguish systematic multi-mappers (used to define communities of aggregated features) from residual multi-mappers (ignored in feature aggregation and quantified in a weighted manner) prior to aggregation. We believe that the graph provides an integrative representation of transcripts with multi-locus profiles and that it enhances interpretability of results. In our results, we found the sub-graphs for each small-RNA biotype had different topologies, linked to its biogenesis and nature. Repeated-loci structure of some well-know transcripts is already incorporated in the gene symbol (e.g. MIR9-1, MIR9-2, MIR9-3 annotations for Human MIR9). However, this is not available for all annotations. Already in the 4 model-organisms analysed, we identified similarity patterns in computationally predicted annotations and dissimilarity patterns between different small RNA pseudocopies. Given computationally predicted annotations without detailed information abound in non-model organisms, we believe their RNA-seq based analysis can largely benefit from the MGcount automatic framework. This will improve quantification and will provide an exploratory tool to identify repeated patterns’ structure in annotations through the multi-mapping graph analysis.

MGcount has two main limitations. First, since MGcount defines communities in a data-driven manner, different datasets with different expression patterns will identify a distinct set of communities. Thus, to compare community-level quantification across samples, all the samples need to be quantified in the same run. Secondly, MGcount depends on a GTF and does not perform de-novo annotation. Consequently, it can only quantify what it is annotated. Part of the non-coding transcriptome is still undiscovered and unannotated, even in well-studied species [54]. Yet, in the small-RNA research field, algorithms have been developed to predict and annotate transcript loci based on RNA conservation [55–58]. With the means to computationally annotate regulatory small RNA, MGcount fills a gap by revealing the structure of predicted annotations from experimental evidence coming from RNA-seq data. We envision MGcount’s applicability not only in expression quantification but also in the uncovering of small-RNA genomic structure profiles.

## Conclusion

MGcount is a novel RNA-seq quantification tool that combines two strategies to quantify ambiguous alignments in an adaptive, data-driven manner. Its framework allows a wider and more inclusive interrogation of total-RNA-seq data, incorporating the simultaneous quantification of coding and non-coding transcripts. MGcount models alignment ambiguities with biotype-specific graphs that are used for the detection of communities of sequence-similar transcripts. Besides the quantification of transcript expression, such graphs constitute a powerful computational tool for the inspection of the structure of multi-loci copies from sequencing data, enhancing the interpretability of results. Given its capacity to simultaneously quantify all biotypes and to handle multi-mappers and multi-overlappers, we believe MGcount will contribute to improving the study of protein-coding and regulatory RNAs interplay by means of total-RNA-seq, even in less characterized species, at both bulk and single-cell resolution.

### Additional file

**Additional file 1.** Supplementary figures. a–d Frequency of annotated transcriptomic features overlapping in genomic origin by biotype and organism for Human (a), Arabidopsis (b), Mouse (c) and Nematode (d). Dotplot presents combinations of two (blue) or three (purple) overlapping features of different biotypes whose occurrence exceeds the 5% of the total number of features from the less abundant biotype in the combination. The top barplot shows the log10 of the total number of cases per combination. The right barplot shows the relative proportion of features overlapping with any other feature by biotype. e Proportion of reads from a human total-RNA-seq library overlapping to 1, 2 or more annotations according to raw alignments assignation (left) and after MGcount assignation by hierarchical rounds (right). f Proportion of reads multi-mapping to a given number of genomic locations (up to 50), according to raw alignments assignation (left) and after MG community aggregation by MGcount (right).(HBR: Human Brain, sNC: small non-coding; lNC: long non-coding)). g Comparison of counts when only uniquely-mapping reads are counted; all alignments are counted, all alignments are fractionally counted as 1 divided by the number of multi-assigments or quantified with MGcount. h–j Three random sub-graphs of 500 features after aggregation extracted from the long-RNA graph. Each vertex is an annotated feature. Its size is proportional to its number of aligned reads. Vertices are colored in blue for protein-coding, yellow for pseudogenes and pink for other lncRNA transcripts. Each edge connects two features with shared multi-mappers with thickness proportional to the fraction of shared multi-mappers over the total alignments. Shared grey areas delineate MG communities. k Comparison of the number of features detected by biotype with a mean count of 5 over human brain replicates. Intronic counts for MGcount are not considered. In addition to ambiguous alignment quantification approaches, softwares differ in assignation criteria: RSEM uses a probabilistic criteria; featureCounts and MGcount were configured with the same criteria defined as a full-overlap between all the nucleotides of a read and the annotation; Coco and mmquant equire a minimum number of nucleotides for assignation, which were set according to default parameters (10nt for Coco and 1nt for mmquant). The comparison is made at community-level, where mmquant merged genes are annotated here as mmquant communities; and at gene-level, where for communities algorithms a gene is detected if it belongs to a detected community. MGcount results in a lower number of features since each community is quantified as a single feature. In addition, the communities approach allows a more inclusive quantification of the individual features collapsed in communities and facilitates multi-mapping reads back-trace in case of interest, as compared to other methods. Incorporating annotations from multiple sources in the custom GTF allows to detect more transcripts and biotypes. Mmquant quantification leads to a very large number of communities since the same gene can be part of multiple merged genes, which can difficult differential feature expression analysis, as opposed to MGcount.

## Data Availability

The compiled software and the source code are available at the GitHub repository of MGcount: https://github.com/hitaandrea/MGcount. The bulk-cell RNA-seq datasets generated and analysed during the current study are available in the following link https://filedn.com/lTnUWxFTA93JTyX3Hvbdn2h/mgcount/rawdata.zip.”. The single-cell RNA-seq dataset analysed during the current study is available in the NCBI Gene Expression Omnibus (GEO), under the accession GEO: GSE151334.

## Appendix

### Methods

#### Samples origin

Human brain total-RNA was commercially acquired (Ambion, AM7962). K562 cells (clone CCL-243) were cultured with IMDM (ATCC - 30-2005 and 10% FBS, Hyclone - SH30071.03HI) and total-RNA was extracted and purified with the Qiagen miRNeasy Mini kit (cat no. 217004) according to the user manual.* Arabidopsis thaliana* hydroponic plants were grown and harvested after 10 weeks and total-RNA was extracted and purified according to protocol [59] in the botanical lab at the University of Liége. Total-RNA from snap frozen mouse liver tissue (P62 overexpressing mice grown and euthanized after 5 weeks) and from Nematode (3000 *C. elegans* N2 wildtype worms per sample) was isolated by immediate lysis in TriReagent (Sigma-Aldrich, Seelze, Germany), purified with acid phenol and additionally digested with DNAseI (Invitrogen, Karlsruhe, Germany) by Martin Simon and his team at the Molekulare Zellbiologie und Mikrobiologie der Bergischen Universität Wuppertal.

#### Libraries preparation and sequencing

Libraries for *arabidposis* roots, mouse liver, nematode, human brain and K562 human cells were prepared in duplicates with the D-Plex Small RNA-seq Kit for Illumina (Diagenode Cat#C05030001) which employs polyA tailing and template-switching. Further, two additional libraries for each human template (human brain and K562 cells) were prepared after enriching for small-RNA content with MiRNeasy tissue/cells advanced kit (Qiagen Cat#217684) for the quantification validation experiment. Sequencing was performed with Illumina technology under the following parameters: SE75 for human brain, SE50 for K562 cells samples and SE100 for the rest of samples (nematode, arabidopsis and mouse). All the libraries were prepared and sequenced in duplicates.

#### NGS pre-processing

Unique molecular identifiers (UMI) of length 12 were extracted from each read with fumi_tools v.0.18.1. Reads were trimmed for Illumnina adapters and polyA tails using cutadapt v3.0 with arguments “–trim-n –match-read-wildcards -u 16 -n 4 -a AGATCGGAAGAGCACACGTCTG -a AAAAAAAA -a GAACTCCAGTCAC -e 0.2 –nextseq-trim 20 -m 15” and subsequently aligned to the reference genomes using STAR v2.7.0d with arguments—outFilterMultimapScoreRange 0 –outFilterMultimapNmax 50 –outFilterMismatchNoverLmax 0.05 –outFilterMatchNmin 15 –outFilterScoreMinOverLread 0 –outFilterMatchNminOverLread 0 –alignIntronMax 1 –readFilesCommand zcat –outSAMtype BAM SortedByCoordinate –outSAMunmapped Within”. Finally, PCR clones were removed by UMI-based deduplication using fumi_tools.

#### qPCR experiment

A subsequence from 20 different small-RNA targets was selected to design a miRCURY LNA custom PCR plate of 96 wells (Cat #339330) so that each plate contained 4 wells measuring the same target. Two plates were employed to quantify targets expression in quadruplicates for human brain and K562 samples. Relative abundance of each target was estimated by normalizing Cq values obtained with RT-qPCR for each target with respect to SNORD49A.

#### Database integration

To generate the GTF files integrating annotations from multiple databases Ensembl was considered the primary annotations source. Next, annotations from different databases were appended after curation and reformatting to an Ensembl-like structure. piRNA and siRNA were reformated as three GTF rows encoding to an Ensembl-like single gene-transcript-exon structure. miRNA precursor and tRNA features were reformatted to Ensembl-like gene entries while the miRNA mature features and tRNA fragments [tRF] were integrated as transcript and exon features with gene_id and gene_name given by its precursor. Since RNACentral aggregates multiple annotation sources, annotations from this database (piRNA in mouse and siRNA in arabidopsis) were semi-automatically curated with a custom script detecting groups of annotations overlapping by more than half of the annotation body (considered redundant) and selecting the annotation with median coordinates within the overlapping set for integration. The full script to generate the 4 GTF files is available in the MGcount Github repository. According to the specie, annotations include: *A. thaliana*: Ensembl, miRBase (microRNA) and RNACentral (siRNA); *H. Sapiens * Ensembl, DASHR (piRNA and tRF) and miRBase (microRNA); *M. musculus*: Ensembl, miRBase (microRNA) and RNAcentral (piRNA); *C. elegans*: Ensembl and miRBase (microRNA).

#### Single cell total-RNA-seq data analysis

Single-cell dataset count tables were pre-processed with the Seurat package [60]. Cells with more than 2000000 counts or less than 2000 features in the reference pipeline [14] were filtered out from both reference table and MGcount table. Counts were log-normalized with a scale factor of 1000000; centered and scaled by the vector of variable features detected with the variance stability transformation method with default parameters. Principal Component Analysis was performed on the subset of small-RNA and long-RNA exonic counts. First and second Principal Components were employed for visualization (Fig. 5a). Deferentially expressed small-RNA features were detected with a Wilcoxon Rank Sum test (adjusted p-value below 0.05) between cell populations.

## Availability and requirements

Project name: MGcount

Project home page: https://github.com/hitaandrea/MGcount

Operating system(s): GNU/Linux

Other requirements: featureCounts

Programming language: Python3

License: GNU GPL

Any restrictions to use by non-academics: None

## Abbreviations

BAM: Binary sequence alignment/map (SAM) format
GTF: Gene transfer format
lncRNA: Long non-coding RNA
mRNA: Messenger RNA
tRF: tRNA fragment
HGNC: Human Gene Nomenclature Committee
