# Correction: MGcount: a total RNA-seq quantification tool to address multi-mapping and multi-overlapping alignments ambiguity in non-coding transcripts

**DOI:** 10.1186/s12859-022-04725-8

**Published:** 2022-06-01

**Authors:** Andrea Hita, Gilles Brocart, Ana Fernandez, Marc Rehmsmeier, Anna Alemany, Sol Schvartzman

**Affiliations:** 1 Epigenetics Unit, Diagenode s.a., Liège, Belgium; 2 Department of Biology, Humboldt-Universität Zu Berlin, Berlin, Germany; 3 Department of Anatomy and Embryology, Leiden University Medical Centre, Leiden, The Netherlands

## Correction to: BMC Bioinformatics (2022) 23:39 https://doi.org/10.1186/s12859-021-04544-3

Following the publication of the original article [1], the authors identified an error in Fig. 2 and caption 2c. The correct figure is given below, and the caption has been updated from ‘’Reads ri (i = 1, 10)’’ to ‘’ Reads ri (i = 1, 11).’’

**Fig. 2:**
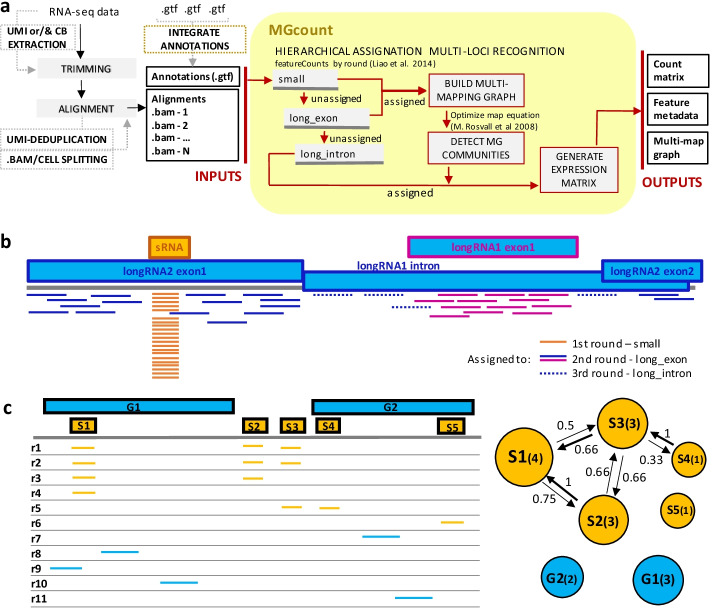
MGcount strategy. **a** MGcount takes a set of genomic alignments (BAM files) and a GTF RNA feature annotations file as inputs. The algorithm assigns reads hierarchically and then models multi-mapping assignments in a graph using the Rosvall’s map equation [36, 37]. As output, MGcount provides an RNA expression count matrix (where feature communities are collapsed as new defined features), a feature metadata table and the graphs. **b** Illustration of how the hierarchical assignation can resolve multi-overlappers: reads that map to small-RNA and long-RNA features are assigned to small-RNA in the first round; reads that map to long-RNA introns and long-RNA exons are assigned to long-RNA exons in the second round; remaining reads are assigned in the last round. **c** Illustration of multi-mapping small-RNA and long-RNA exon graphs generation by MGcount. Reads ri (i = 1, 11) have been hierarchically assigned to *S*_1_, *S*_2_, *S*_3_, *S*_4_, *S*_5_ (small-RNA biotypes, yellow), and *G*_1_, *G*_2_ (long-RNA biotypes, blue). Each vertex in the directional multi-mapping graphs (right) corresponds to a feature and has a size proportional to the logarithm of the number of alignments. Edges connect vertices with common multi-mapping reads, with weights proportional to the number of common multi-mappers normalized by the total number of alignments of the source vertex. Hence, the weight of the edge connecting S1 with S2 becomes 3/4 (reads mapping both S1 and S2 divided by reads aligned to S1). (CB: Cell Barcode, UMI: Unique Molecular Identifier)

The original article [1] has been corrected.
