# Supplementary material for: MGcount: a total RNA-seq quantification tool to address multi-mapping and multi-overlapping alignments ambiguity in non-coding transcripts
Source: BMC Bioinformatics. 2022 Jan 14;23:39. doi: 10.1186/s12859-021-04544-3 (PMC8760670; doi:10.1186/s12859-021-04544-3)
Supplement: Supplementary file 1 — Additional file 1. Supplementary figures. a–d Frequency of annotated transcriptomic features overlapping in genomic origin by biotype and organism for Human (a), Arabidopsis (b), Mouse (c) and Nematode (d). Dotplot presents combinations of two (blue) or three (purple) overlapping features of different biotypes whose occurrence exceeds the 5% of the total number of features from the less abundant biotype in the combination. The top barplot shows the log10 of the total number of cases per combination. The right barplot shows the relative proportion of features overlapping with any other feature by biotype. e Proportion of reads from a human total-RNA-seq library overlapping to 1, 2 or more annotations according to raw alignments assignation (left) and after MGcount assignation by hierarchical rounds (right). f Proportion of reads multi-mapping to a given number of genomic locations (up to 50), according to raw alignments assignation (left) and after MG community aggregation by MGcount (right).(HBR: Human Brain, sNC: small non-coding; lNC: long non-coding)). g Comparison of counts when only uniquely-mapping reads are counted; all alignments are counted, all alignments are fractionally counted as 1 divided by the number of multi-assigments or quantified with MGcount. h–j Three random sub-graphs of 500 features after aggregation extracted from the long-RNA graph. Each vertex is an annotated feature. Its size is proportional to its number of aligned reads. Vertices are colored in blue for protein-coding, yellow for pseudogenes and pink for other lncRNA transcripts. Each edge connects two features with shared multi-mappers with thickness proportional to the fraction of shared multi-mappers over the total alignments. Shared grey areas delineate MG communities. k Comparison of the number of features detected by biotype with a mean count of 5 over human brain replicates. Intronic counts for MGcount are not considered. In addition to ambiguous alignment quantification approach [file 12859_2021_4544_MOESM1_ESM.pdf]

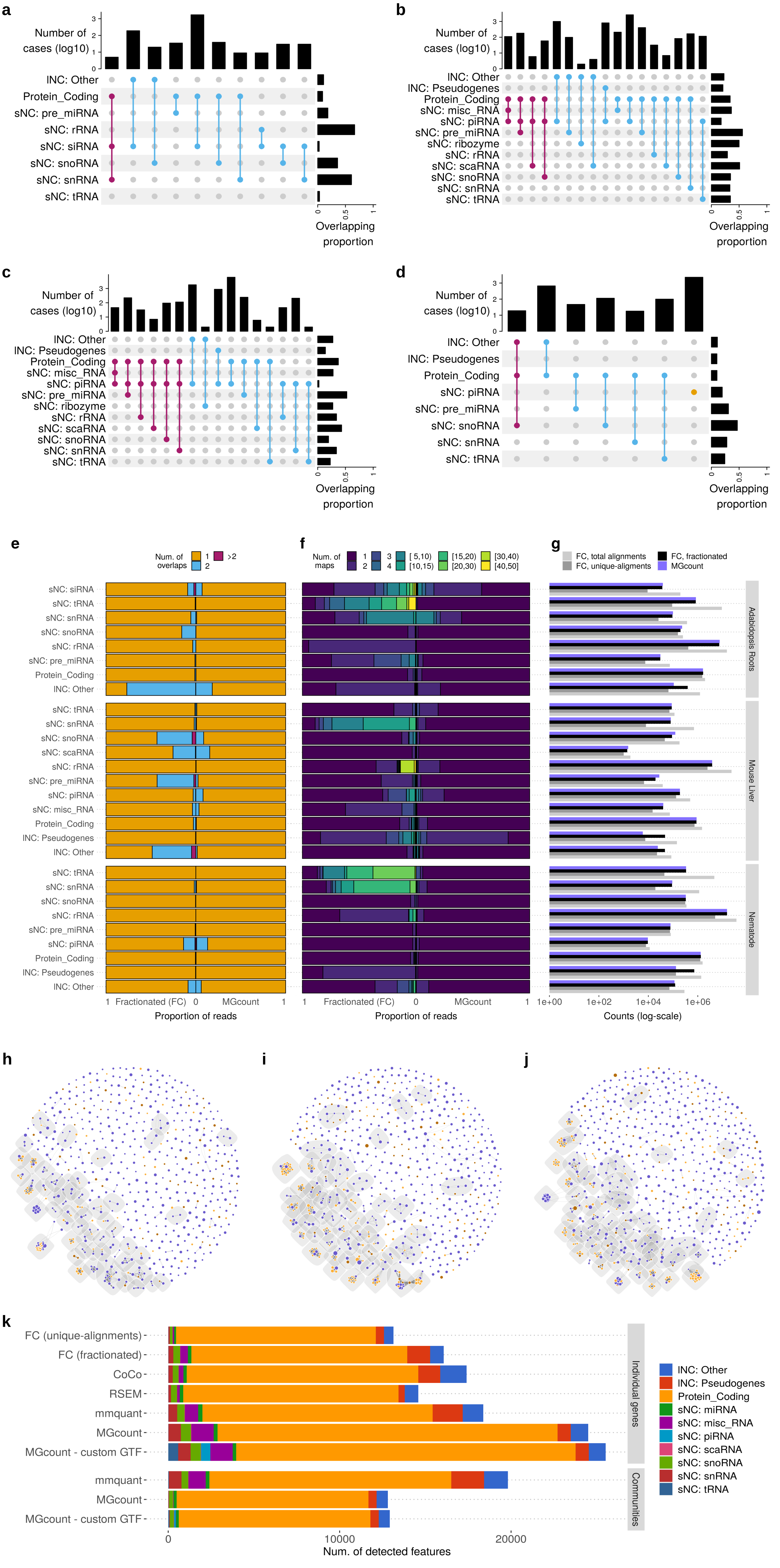
a
Number of cases (log10)
INC: Other
Protein_Coding
sNC: pre_miRNA
sNC: rRNA
sNC: siRNA
sNC: snoRNA
sNC: snRNA
sNC: tRNA
Overlapping proportion
b
Number of cases (log10)
INC: Other
INC: Pseudogenes
Protein_Coding
sNC: misc_RNA
sNC: piRNA
sNC: pre_miRNA
sNC: ribozyme
sNC: rRNA
sNC: scaRNA
sNC: snoRNA
sNC: snRNA
sNC: tRNA
Overlapping proportion
c
Number of cases (log10)
INC: Other
INC: Pseudogenes
Protein_Coding
sNC: misc_RNA
sNC: piRNA
sNC: pre_miRNA
sNC: ribozyme
sNC: rRNA
sNC: scaRNA
sNC: snoRNA
sNC: snRNA
sNC: tRNA
Overlapping proportion
d
Number of cases (log10)
INC: Other
INC: Pseudogenes
Protein_Coding
sNC: piRNA
sNC: pre_miRNA
sNC: snoRNA
sNC: snRNA
sNC: tRNA
Overlapping proportion
e
Num. of overlaps 1 2 >2
f
Num. of maps 1 2 3 4 [5,10) [10,15) [15,20) [20,30) [30,40) [40,50]
g
FC, total alignments
FC, unique-aligments
FC, fractionated
MGcount
Adabidopsis Roots
Mouse Liver
Nematode
Fractionated (FC) 0 MGcount
Proportion of reads
Counts (log-scale)
h
i
j
k
FC (unique-alignments)
FC (fractionated)
CoCo
RSEM
mmquant
MGcount
MGcount - custom GTF
Individual genes
Communities
INC: Other
INC: Pseudogenes
Protein_Coding
sNC: miRNA
sNC: misc_RNA
sNC: piRNA
sNC: scaRNA
sNC: snoRNA
sNC: snRNA
sNC: tRNA
Num. of detected features

Supplementary figures. a-d Frequency of annotated transcriptomic features overlapping in genomic origin by biotype and organism for Arabidopsis (a), Human (b), Mouse (c) and Nematode (d). Dotplot presents combinations of one (orange), two (blue) or three (purple) overlapping features of different biotypes whose occurrence exceeds the 5% of the total number of features from the less abundant biotype in the combination. The top barplot shows the log10 of the total number of cases per combination. The right barplot shows the relative proportion of features overlapping with any other feature by biotype. **e** Proportion of reads from a human total-RNA-seq library overlapping to 1, 2 or more annotations according to fractionated alignments assignation (left) and after MGcount assignation by hierarchical rounds (right). **f** Proportion of reads multi-mapping to a given number of genomic locations (up to 50), according to fractionated alignments assignation (left) and after MG community aggregation by MGcount (right).(HBR: Human Brain, sNC: small non-coding; lNC: long non-coding)). **g** Comparison of counts when only uniquely-mapping reads are counted; all alignments are counted, all alignments are fractionally counted as 1 divided by the number of multi-assigments or quantified with MGcount. **h-j** Three random sub-graphs of 500 features after aggregation extracted from the long-RNA graph. Each vertex is an annotated feature. Its size is proportional to its number of aligned reads. Vertices are colored in blue for protein-coding, yellow for pseudogenes and brown for other lncRNA transcripts. Each edge connects two features with shared multi-mappers with thickness proportional to the fraction of shared multi-mappers over the total alignments. Shared grey areas delineate MG communities. **k** Comparison of the number of features detected by biotype with a mean count over 5 over human brain replicates. Intronic counts for MGcount are not considered. In addition to ambiguous alignment quantification approaches, softwares differ in assignation criteria: RSEM uses a probabilistic criteria; featureCounts and MGcount were configured to require a full-overlap between the read and the annotation; Coco and mmquant require a minimum number of nucleotides for assignation, which were set according to default parameters (10nt for Coco and 1nt for mmquant). The comparison is made at community-level, where mmquant merged genes are annotated here as mmquant communities; and at gene-level, where for communities algorithms a gene is detected if it belongs to a detected community. MGcount results in a lower number of features since each community is quantified as a single feature. In addition, the communities approach allows a more inclusive quantification of the individual features collapsed in communities and facilitates multi-mapping reads back-trace in case of interest, as compared to other methods. Incorporating annotations from multiple sources in the custom GTF allows to detect more transcripts and biotypes. mmquant quantification leads to a very large number of communities since the same gene can be part of multiple merged genes, as opposed to MGcount.
